# Case-Based Perspectives on the Management of Genitourinary Syndrome of Menopause

**DOI:** 10.3390/clinpract16030060

**Published:** 2026-03-12

**Authors:** Jissy Cyriac, Richa Sood

**Affiliations:** 1 Menopause and Women’s Sexual Health Clinic, Division of General Internal Medicine, Mayo Clinic, Rochester, MN 55905, USA; cyriac.jissy@mayo.edu; 2 Division of Community Internal Medicine, Geriatrics, and Palliative Care, Mayo Clinic, Rochester, MN 55905, USA

**Keywords:** atrophic vaginitis, dyspareunia, genitourinary syndrome of menopause, menopause, vulvovaginal atrophy, breast cancer, postmenopausal women, sexual dysfunction

## Abstract

Background and Objectives: Genitourinary syndrome of menopause (GSM), previously known as vulvovaginal atrophy, is a chronic, progressive hypoestrogenic condition affecting vulvovaginal, urinary and sexual health in women. Common symptoms include vaginal dryness, itching, dyspareunia, urinary urgency and recurrent urinary tract infections (UTIs). Despite the high prevalence, GSM is underdiagnosed and undertreated, thereby negatively impacting women’s quality of life. To illustrate the practical aspects of GSM diagnosis and provide evidence-based management, we present a case-based narrative review synthesizing recently published, high-quality evidence. Materials and Methods: Evidence was drawn from multiple sources through targeted searches of databases, and included the 2025 AUA/SUFU/AUGS guideline (AUA), the 2024 NICE network meta-analyses (NICE), a 2025 systematic review/meta-analysis in breast-cancer survivors, the 2020 Menopause Society GSM Position Statement, the 2018 NAMS/ISSWSH breast cancer consensus, several primary source citations and other high quality peer-reviewed publications. Results: Five illustrative composite case vignettes of GSM are presented to highlight the evaluation strategy and evidence-supported treatment choices. Nonhormonal options are the first line treatments for mild GSM symptoms, either with or without the addition of vaginal estrogen therapy. For moderate to severe GSM, low-dose vaginal estrogen, vaginal DHEA, and ospemifene are all effective FDA-approved options. In breast cancer survivors, individualized decisions with oncology input are warranted. Maximal caution and a shared decision-making approach is required for women using Aromatase Inhibitors (AIs) for breast cancer risk reduction when choosing treatments for GSM. Conclusions: Treating GSM improves vaginal, sexual and urinary outcomes and quality of life of women. Clinicians need to proactively screen for GSM and offer evidence-based treatment options. The treatment decisions in breast cancer survivors are nuanced, requiring a shared-decision approach.

## 1. Introduction

Genitourinary syndrome of menopause (GSM) describes a spectrum of vulvovaginal, urinary, and sexual symptoms caused by estrogen deficiency. The term GSM has replaced the older term atrophic vaginitis to better reflect the multisystem nature of the condition and its hormonal basis [1]. GSM affects an estimated 50–70% of menopausal women, although population studies suggest even higher prevalence when mild symptoms are included [2,3]. GSM is progressive and does not remit spontaneously.

Despite its high prevalence, it remains underdiagnosed and undertreated, leading to significant negative consequences for quality of life, sexual satisfaction, intimate relationships, and genitourinary health [2,3]. Acknowledging the significant impact of GSM on a woman’s comfort, relationships, and self-image is crucial. Since many women mistakenly believe these changes are an unavoidable consequence of aging, clinician guidance that reframes GSM as a treatable medical condition, and outlines effective options, encourages participation in shared, informed decisions [3,4].

Management of GSM encompasses both hormonal and nonhormonal approaches. Hormonal therapies include low-dose vaginal estrogen, intravaginal dehydroepiandrosterone (DHEA), and the oral selective estrogen receptor modulator (SERM), ospemifene. Nonhormonal options consist of vaginal lubricants and moisturizers, complemented by adjunctive interventions such as pelvic floor physical therapy, dilator therapy and regular sexual activity to maintain tissue health.

Using clinical case examples, this manuscript will outline practical, evidence-based approaches toward the diagnosis and management of GSM in average risk patients (Case 1), in breast cancer survivors (Cases 2a, 2b and 2c), and in menopausal women with urinary tract infections (UTIs) (Case 3).

## 2. Materials and Methods

This article was developed as a narrative review intended to provide a clinically focused synthesis of current evidence and practice recommendations for the management of GSM. The review was not designed as a systematic review and does not follow PRISMA methodology.

Evidence informing this review was identified through targeted searches of PubMed and Google Scholar, with prioritization of high-quality randomized clinical trials, large observational studies, and current international clinical practice guidelines, including those from the American Urological Association/Society of Urodynamics, Female Pelvic Medicine & Urogenital Reconstruction/American Urogynecologic Society (AUA/SUFU/AUGS), The Menopause Society, and the National Institute for Health and Care Excellence (NICE). The majority of the clinical outcome studies were published within the past decade, reflecting current clinical practice and evolving evidence. Searches were supplemented by manual review of references from key guideline documents and landmark publications.

The literature identification process was selective rather than exhaustive, consistent with a narrative review approach. No formal inclusion or exclusion criteria, predefined sample-size thresholds, risk-of-bias assessments, or systematic screening procedures were applied. The goal was to contextualize clinically relevant evidence and highlight areas of consensus, uncertainty, and evolving practice.

## 3. Case-Based Discussions for GSM

The 5 cases presented are composite clinical vignettes derived from common scenarios encountered in routine clinical practice. They are intended to illustrate diagnostic considerations, therapeutic decision-making, and guideline-informed management strategies for GSM and do not represent individual patient case reports.

**Case 1:** GSM in a Low-Risk Postmenopausal Woman.History of present illness
58-year-old, postmenopausal female, G2P2Last menstrual period (LMP) at age 50 yearsPresents with a three-year history of progressive vaginal dryness, itching, irritation, and insertional dyspareunia leading to a decline in her libido and sexual frequency
Past history
HypertensionHyperlipidemiaUp to date on breast, cervical, and colon cancer screenings
Medications & supplements
Lisinopril 20 mg orally once dailyMultivitamin one tablet orally once dailyCalcium 600 mg orally twice dailyVitamin D3 400 IU orally twice daily
Social history
Alcohol use—5 oz of wine per weekNon-smokerNo recreational drug useMarried, in a supportive relationship with her 60-year-old husband
Gynecologic examinationMirrored exam performed with patient participation. External genitaliaSome loss of labial bulkNo fissuring or white lesions seen in the interlabial folds or perineumSpeculum examinationPale, thin introitus and vaginal mucosa with loss of rugae.Light physiologic appearing secretions noted with no odorCervix visualized and healthyBimanual examinationElicits mild insetional discomfort due to introital narrowing and thinningThe uterus is normal in size; non-tenderNo cervical motion tenderness or masses

Diagnosis:

Based on her menopausal status, characteristic symptoms (dryness, irritation, dyspareunia), and exam findings (loss of labial bulk, diminished rugae, introital narrowing, pallor), a diagnosis of GSM is made. Laboratory testing or pH measurement or vaginal maturation index are not required when the presentation is classic. Differential diagnoses include infectious or inflammatory vaginitis in appropriate situations, and vulvar dermatoses should be ruled out if clinically suspected [1,4].

Management:

This case introduces the fundamental principles of GSM management, including patient education, counseling, and nonhormonal approaches, in a low-risk postmenopausal woman. These core elements also underpin the management strategies applied in subsequent cases involving breast cancer survivors.

*Education and Counseling:* Incorporating a mirrored examination allows clinicians to visually demonstrate the anatomic changes associated with GSM while simultaneously providing patient education. A handheld mirror during pelvic examination has been shown to enhance patients’ understanding of their anatomy and to foster a greater sense of control and engagement in their care [5]. Use of easy-to-understand patient educational materials on GSM can help ensure patients understand the array of treatment options available to them for management.

Women with GSM frequently encounter sexual health concerns. Thus, equally important is using open-ended questions about sexual health and function, and validation of those concerns [6]. The PLISSIT (Permission, Limited Information, Specific Suggestions, Intensive Therapy) framework offers a practical approach to sexual health assessment and counseling [7], while validated tools such as the Female Sexual Function Index (FSFI) can be used before visits to efficiently identify key concerns when time is limited [8].

*Nonhormonal treatments:* Gentle, hypoallergenic vulvar care and avoidance of irritating or fragranced products are recommended as part of best practices in GSM management. These practices complement hormonal and nonhormonal therapeutic interventions by minimizing further irritation of the sensitive vulvovaginal tissues.

***Lubricants and moisturizers***: Vaginal lubricants and moisturizers are recommended as first-line treatments for GSM, either alone or concurrently with vaginal hormone therapy [4,9]. Lubricants are recommended for application during sexual activity to either or both partners’ genitals to help reduce friction and discomfort. Moisturizers, on the other hand, need to be used consistently, outside of sexual activity, to restore vaginal tissue hydration and elasticity. Randomized trials and meta-analyses have not shown clear superiority of one product over another, so personal preference guides selection [4,10].

When recommending nonhormonal therapies for GSM, the physiologic pH and osmolality of the product are critical for mucosal safety and efficacy (Table 1). In healthy premenopausal women, vaginal pH typically ranges from 3.8 to 4.5, supporting a lactobacilli-dominant microbiota that protects against infection. Postmenopausal hypoestrogenism raises vaginal pH, predisposing those affected to dysbiosis and mucosal irritation. Therefore, vaginal moisturizers and lubricants formulated with a pH approximating 3.5 to 5.0 help restore an acidic environment conducive to epithelial health. Equally important is osmolality: products that are markedly hyperosmolar can draw water from epithelial cells, leading to dehydration, irritation, and microtrauma. The World Health Organization recommends an osmolality below 1200 mOsm/kg, and ideally near the physiologic range of 280–310 mOsm/kg, to minimize epithelial toxicity and preserve mucosal integrity [11]. Clinicians should therefore guide patients toward formulations that are both pH-balanced and iso-osmolar, avoiding hyperosmolar or alkaline products that may exacerbate GSM symptoms or compromise the vaginal microbiome [12]. Vaginal lubricants and moisturizers will be discussed in more detail in case 2.

*Hormonal treatments*: These include low-dose vaginal estrogen products, intravaginal dehydroepiandrosterone (DHEA) inserts, and oral ospemifene. Systemic estrogen therapy, when used for appropriate indications, may help GSM symptoms, although low-dose vaginal estrogen (LDVE) has a more favorable risk profile than systemic ET because estrogen doses are significantly lower and systemic absorption is minimal [4].

***Vaginal estrogen***: LDVE is highly effective for the treatment of GSM and provides adequate symptom relief while maintaining minimal systemic absorption, particularly after vaginal epithelial thickness is restored [4,9]. Progestogen co-therapy is not required [4]. Multiple randomized controlled trials of LVDE formulations demonstrate symptom improvement within 2–4 weeks, maximal benefit by approximately 12 weeks, and postmenopausal-range serum estradiol levels (<20 pg/mL) [13,14,15,16].

A variety of vaginal estrogen formulations, including creams, tablets, softgels, and rings are all FDA-approved for GSM (Table 2). These preparations appear to have comparable efficacy [17], and the selection is guided by provider experience, patient preference and insurance coverage. Some specific features of these formulations may also guide the treatment decisions. For example, estrogen creams provide the option of massaging it directly over the vulvar vestibule and in the lower 1/3rd of vagina (avoiding the traditional applicator method). However, the disadvantage with creams is an inability to dose to an exact amount, while the premeasured vaginal tablets and softgels offer that advantage. For women unwilling or unable to use these vaginal products that require twice weekly insertion or application, a low-dose vaginal estrogen ring is a suitable option as it requires replacement after 90 days. Consistent use of any of these options is effective for GSM. Education regarding the progressive nature of GSM without treatment and the importance of consistent treatment for symptom relief is essential to support adherence and improve long-term outcomes [4].

The boxed warnings historically included in FDA package inserts for LDVE products represent class labeling applied to systemic estrogen therapies [18]. However, LDVE formulations are associated with minimal systemic absorption and a substantially more favorable safety profile [4]. Although the clinical trial safety data is limited to one year [4], large cohort studies have shown no increase in the risk of breast or endometrial cancer or cardiovascular events with LDVE use among average-risk women [19,20]. Following expert review and to better align labeling with current evidence regarding the benefits and risks of hormone therapy, the FDA has announced the removal of boxed warnings from all estrogen products, including systemic formulations, further reinforcing the robust safety data supporting the use of LDVE [21].

***Vaginal DHEA***: Vaginal DHEA (prasterone) is an FDA-approved local therapy for GSM, available as a 6.5 mg vaginal insert administered once daily at bedtime. Endogenous DHEA is an inactive sex-steroid precursor physiologically secreted by the adrenal glands that undergoes intracellular conversion to active androgens and estrogens within peripheral tissues through enzymatic pathways involving 17β-hydroxysteroid dehydrogenase and aromatase [22]. When exogenous DHEA is administered intravaginally, it is absorbed by the vaginal epithelium where it converts to testosterone and estradiol. Vaginal DHEA results in significant improvements in vaginal cytology, pH, vaginal dryness and dyspareunia. At the dose of 6.5 mg, serum estradiol and testosterone levels remain within the postmenopausal range, and endometrial safety has been demonstrated up to 52 weeks of follow-up. With this favorable safety profile and evidence of efficacy, vaginal DHEA is FDA-approved for GSM treatment [23,24,25] and is supported by multiple guidelines as an effective non-estrogenic option for moderate-to-severe GSM [4,9].

***Ospemifene***: Ospemifene is an oral SERM with tissue-selective estrogen agonist and antagonist activity. Ospemifene exhibits estrogenic effects on vulvovaginal tissues while maintaining anti-estrogenic activity in breast tissue. It is FDA-approved for the treatment of moderate-to-severe GSM at a dose of 60 mg once daily [4,26]. Multiple clinical trials have demonstrated both the safety and efficacy of ospemifene for the treatment of GSM [27]. In a multicenter, double-blind, randomized controlled trial involving 605 postmenopausal women with vulvovaginal atrophy, ospemifene produced significant improvements in vaginal cytology, pH, dyspareunia, and overall sexual function compared with placebo after 12 weeks of therapy [28]. Safety assessments from this and subsequent trials showed no increase in thromboembolic events, endometrial hyperplasia, or endometrial carcinoma with ospemifene use [28,29]. Furthermore, a comprehensive meta-analysis of 44 studies including 12,637 participants confirmed that ospemifene’s efficacy and safety profiles are comparable to those of other established hormonal and nonhormonal therapies for GSM [29]. Ospemifene is supported by guidelines as a safe and effective treatment option for moderate-to-severe GSM symptoms [4,9].

*Ancillary therapies:* Pelvic floor physical therapy incorporating pelvic floor relaxation exercises can help maintain vaginal blood flow and elasticity [30]. Use of medical vibrator therapy can also be a helpful adjunct for improved blood flow [31].

*Energy-based therapies:* A detailed discussion of these therapies are outside the scope of this review. In brief, energy-based devices such as fractional CO_2_ or Er: YAG lasers are not recommended outside clinical controlled trials due to insufficient evidence of safety and lack of demonstrable benefits over the current standard of care [4,32].

Outcome:

The patient chose to start estradiol 0.01% vaginal cream. She was instructed to apply it in a dose of 1 g per application every day for two weeks, followed by the maintenance regimen of 1 g twice weekly. She was instructed to apply the cream using her finger rather than the applicator, gently massaging it over the introitus and lower vaginal tissues for several seconds to enhance absorption, improve adherence, and minimize messiness or leakage. She also chose a long-acting, hypoallergenic, water-based lubricant to be used during sexual activity for additional comfort. At her three-month follow-up visit, she reported marked improvement in vaginal lubrication, resolution of itching and insertional pain, and restoration of comfortable sexual activity. Ongoing maintenance therapy with low-dose vaginal estrogen cream twice weekly was advised, with periodic follow-up recommended for symptom reassessment and continued monitoring.

### Cases 2a, 2b and 2c: Breast Cancer Survivors with GSM

**Figure 1:**
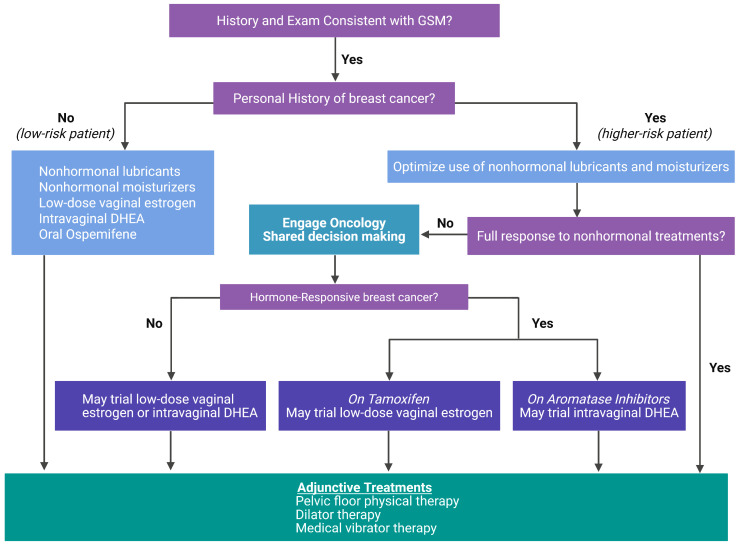
A proposed approach to GSM management.

Breast cancer survivors represent a uniquely affected subgroup, because adjuvant endocrine therapies such as tamoxifen and aromatase inhibitors (AIs) exacerbate estrogen deficiency in urogenital tissues [30]. The hypoestrogenic state results in epithelial thinning, increased vaginal pH, loss of elasticity, and changes in the microbiome, particularly a reduction in Lactobacillus species and an increase in pH-dependent anaerobes [33,34]. In women with a history of breast cancer, GSM symptoms tend to be more severe, and treatment depends on multiple factors, including the breast cancer receptor status, disease extent and time interval since diagnosis, and the type of medications being used to reduce the risk of future recurrence [35]. The treatment goal in this subgroup of women is to alleviate GSM symptoms with an individualized approach after risk–benefit discussion (Figure 1).

**Case 2a:** GSM with a History of Estrogen Receptor (ER) + Breast Cancer; on Treatment with Tamoxifen.History of present illness
48-year-old, postmenopausal, G2P2 femaleLast menstrual period age 47History of breast cancer diagnosed at age 45Presents with vaginal dryness, discharge, dyspareunia
Past history
Stage 1 ER/PR positive invasive ductal cancer diagnosed at age 45 years○Underwent lumpectomy and radiation therapy○Currently on selective estrogen receptor modulator (tamoxifen) for breast cancer recurrence risk reductionUp to date on breast cancer surveillance, colon and cervical cancer screening
Medications & supplements
Tamoxifen 20 mg orally once dailyLevothyroxine 100 μg orally once dailyMultivitamin one tablet orally once dailyTums 600 mg orally twice daily
Social history
Alcohol 1–2 drinks (1.5 oz each) per monthNonsmokerNo recreational drug useMarried, decreased sexual desire, with resulting relational strain with her 51-year-old husband
Gynecologic examinationMirrored exam performed with patient participation. External genitaliaLabial thinning with preserved interlabial foldsNo adhesions or white lesionsMucosal pallor and decreased rugosity noted at the introitusSpeculum examination
Copious clear discharge present with no odor or underlying erythemaDecreased vaginal rugosityCervix visualized and healthyVaginal swabs obtained to rule out infectious etiology of the discharge (and results came negative for infectious vaginitis)Bimanual examination
Performed with two fingers and ample lubricationPositive for discomfort at the introitus as well as with deeper palpation over the pelvic floor muscles globallyUterus normal size and mobile, non-tenderNo cervical motion tenderness or adnexal masses noted

Management:

*Education and Counseling*: Individuals with a history of breast cancer represent a unique population that often requires more comprehensive counseling regarding GSM. Building on the educational and counseling principles outlined in Case 1, breast cancer survivors benefit from additional discussion of available treatment options, mechanisms of action, and the evidence supporting efficacy and safety [30]. This information should be contextualized within the severity of symptoms, impact on quality of life, and individual concerns about breast cancer recurrence. When appropriate, engagement of the oncology care team can further support a multidisciplinary treatment approach aligned with both oncologic and sexual health goals. These counseling principles are also applied in Cases 2b and 2c.

*Nonhormonal treatments:* Vaginal lubricants and moisturizers are the first-line treatments for GSM, particularly in women with breast cancer. Lubricants are used during sexual intimacy, while vaginal moisturizers are applied regularly several times per week, outside of sexual activity [12,30,36]. Lubricants are preferred to be water- or silicone-based. Hypoallergenic, long-acting, paraben-free lubricants, when applied to the vulvovaginal tissues and/or the partner’s genitals can help minimize friction and reduce discomfort during sexual intimacy [4]. Glycerin-based lubricants are not preferred as these may increase the risk of bacterial vaginosis [4,12,37]. Although some women prefer natural oils such as olive or coconut oil for lubrication, these may stain fabrics and lack long-term safety data [4]. Petroleum-based lubricants are discouraged due to an increased risk of bacterial vaginosis and vaginal colonization with *Candida* species [4,38]. Arousing or stimulating lubricants are best avoided, as are additives or warming agents because they may irritate the delicate vaginal mucosa [12]. Lubricants with paraben products are best avoided, as in vitro studies have shown parabens to have weak estrogenic activity which may interfere with cellular signaling pathways [39,40]. Other preservatives such as antibacterial agents and chlorhexidine should also be avoided as these may affect the vaginal microbiome negatively and cause tissue irritation [12,37].

Vaginal moisturizers, owing to their hygroscopic properties, are considered first-line therapy in women with breast cancer [10,30]. When applied intravaginally several times per week, they hydrate newly formed vaginal epithelial cells replacing the older cells, thereby improving the vaginal moisture and pH [12]. Moisturizers are available as gels, suppositories, or liquibeads and are comparable in their efficacy, with no head-to-head studies [4].

Although nonhormonal lubricants and moisturizers are recommended as first-line therapy for GSM, particularly in women with mild symptoms, direct comparative data evaluating their onset of action, durability of symptom relief, and long-term adherence relative to hormonal therapies are limited [4]. In a 12-week, 3-arm randomized clinical trial of 302 women, vaginal moisturizers demonstrated comparable efficacy to low-dose vaginal estrogen and placebo gel in reducing the most bothersome GSM symptoms [38]. Most GSM trials are short-term and do not adequately capture persistence of benefit or real-world adherence. Overall evidence supports that nonhormonal lubricants/moisturizers can provide sufficient relief for many women with mild symptoms, while prescription therapies may offer greater benefit for persistent or moderate–severe symptoms.

*Hormonal treatments:*

***Vaginal estrogen***: Inadequate benefit with nonhormonal therapy may be an acceptable indication to consider LDVE in women on tamoxifen with oncology input and shared decision-making with the patient. Tamoxifen is a selective estrogen receptor modulator (SERM) that acts as an estrogen agonist in the endometrium, and as an antagonist on the estrogen receptors in the breast tissue, thereby reducing the breast-related concerns with LDVE use. Observational studies and large meta-analyses show no increase in breast cancer recurrence or mortality with LDVE in tamoxifen users [41,42,43,44,45]. Contemporary guidelines and consensus statements recommend a stepwise approach of starting nonhormonal treatment with moisturizers and lubricants. For those women experiencing persistent moderate-to-severe symptoms, adding LDVE with premeasured inserts or tablets releasing 4–10 µg of estradiol or with the lowest-dose vaginal estrogen creams is an acceptable strategy [4,10,30].

Observational data demonstrate that low-dose vaginal estrogen does not elevate breast cancer recurrence or mortality in breast cancer survivors [44,45]. A large meta-analyses compiling observational studies found no increased risk for breast cancer recurrence (24,060 patients, odds ratio, 0.48; 95% confidence interval, 0.23–0.98), breast cancer-related mortality (61,695 patients, odds ratio 0.60; 95% confidence interval 0.18–1.95), or overall mortality (odds ratio 0.46; 95% confidence interval 0.42–0.49) [43].

Current guidelines recommend clinical monitoring rather than routine serum estradiol surveillance for women with a history of breast cancer who are using low-dose vaginal estrogen. A short-term 8–12 week follow-up after initiating treatment, followed by annual assessment for clinical response, is an acceptable strategy. No evidence-based duration limits or discontinuation thresholds have been established. Discontinuation is generally considered only in the setting of unexplained vaginal bleeding, lack of clinical benefit, intolerable adverse effects, or a reassessment of risk–benefit balance.

***Vaginal DHEA***: This is another hormonal option available for GSM that improves dyspareunia, vaginal dryness, cytology, pH and sexual health outcomes in randomized trials of postmenopausal women with and without breast cancer [23,24,25,46]. However, vaginal DHEA administration results in slightly increased serum DHEA-S and a testosterone level of unclear clinical significance [47]. Therefore, in the absence of studies with long-term outcome data, the decision to use vaginal DHEA in women with breast cancer on tamoxifen needs to be individualized and based on a shared-decision model.

***Ospemifene***: Ospemifene is contraindicated for use in a woman with estrogen-dependent cancers [48]. The use of ospemifene in breast cancer survivors has not been studied in large randomized controlled trials or directly compared against LDVE. Retrospective analysis of postmenopausal breast cancer survivors in which >1700 women used ospemifene compared to >3400 untreated controls did not demonstrate an increased breast cancer recurrence in the ospemifene group [49]. Prospective studies signal that ospemifene demonstrates efficacy in GSM treatment, but long-term outcome data are lacking [50]. Ospemifene safety has not been studied in women with breast cancer using tamoxifen and should not be used concomitantly in patients on tamoxifen.

*Ancillary therapies*: Pelvic floor relaxation exercises are an important aspect of treatment for the hypertonic pelvic floor muscles contributing toward her symptom of deep dyspareunia. Referral to a pelvic floor physical therapist is the next step in the absence of symptom relief.

Outcome:

The patient started estradiol 10 μg inserts twice weekly, a vaginal moisturizer 3×/week, and a water-based lubricant, as needed. For deeper dyspareunia related to pelvic floor myalgia, pelvic floor relaxation exercises were discussed, including deep slow, diaphragmatic breathing, and ‘Happy Baby Pose’ exercises. The option of visiting with a pelvic floor physical therapist was reviewed for persistent symptoms. Consideration of dilator therapy after pelvic floor muscle relaxation was discussed. The option of visiting with a sex therapist was also reviewed to address the concerns with depressed libido. A follow-up visit was arranged in 3 months to review progress.

**Case 2b:** GSM with a History of ER + Breast Cancer; On Treatment with Aromatase Inhibitors.History of present illness
60-year-old, postmenopausal, G0P0 femaleHistory of breast cancer and currently on aromatase inhibitor therapyPresents with distressing genital symptoms, including burning, itching, and marked vaginal dryness, even when wiping after voidingReports severe dyspareunia that has prevented penetrative sexual activity for more than a year
Past history
HypertensionAnxiety, well-managed with counselingStage IIA ER/PR positive invasive ductal carcinoma 3 years ago○Underwent lumpectomy and radiation therapy, followed by chemotherapy○Currently on anastrozoleLaparoscopic cholecystectomy, 8 years agoUp to date on breast cancer surveillance, colon, and cervical cancer screening
Medication & /supplements
Amlodipine 5 mg orally once dailyAnastrozole 1 mg orally once dailyMultivitamin one tablet once daily
Social history
Rare alcohol use in social settingsNonsmokerNo recreational drug useCommitted relationship with her male partner for the past 12 years
Gynecologic examinationA mirrored examination was performed. External genitaliaScant pubic hair and involution of the labiaNo fissures or white lesionsPale and thin mucosa at vaginal introitusTenderness to light touch using a cotton-tip swab, particularly at the 6 and 12 o’clock positions.Speculum examination:Unable to assess due to severe discomfort despite ample lubricationBimanual examinationPerformed with 1 finger after applying a small amount of 2% lidocaine gel to the sensitive areas of the introitus to minimize discomfortNotable for tightening of the lower vaginaHypertonic and tender pelvic floor muscles diffuselyNo cervical motion tendernessLimited for adnexal assessmentSlight blood-tinging noted on the examining finger

Management:

*Education and Counseling:* As discussed in cases 1 and 2a, it is important to highlight that these symptoms are due to a combination of hypoestrogenic changes in menopause as well as the anti-estrogenic effects of aromatase inhibitors on the estrogen receptor-rich vulvovaginal tissues. Providing reassurance that effective treatments are available to alleviate pain is the next essential component of care.

*Nonhormonal treatments:* The first-line treatments for women with history of ER/PR+ breast cancer on aromatase inhibitor therapy are hypoallergenic long-acting vaginal lubricants and vaginal moisturizers, as well as pelvic floor relaxation exercises and dilator therapy, in appropriate situations. The approach to lubricants and moisturizers mirrors that outlined in Case 2a. In addition, topical lidocaine application to the vulvar vestibule 5–10 min prior to vaginal intercourse, with removal of the excess product before penetration to avoid potential for partner hypoesthesia, can be particularly effective as an adjunct, and it has been shown to reduce insertional dyspareunia significantly compared to placebo in a double-blind RCT of 46 breast cancer survivors [51]. Formulations in 2% gel, 4% aqueous or 5% ointment strengths are commonly used, with the specific choice guided by patient preference, tolerance, and insurance coverage. Use is intermittent rather than daily and is contraindicated in patients with known hypersensitivity to local anesthetics or significant mucosal ulceration. Regular sexual activity, including external stimulation or penetration, if tolerated, can promote genital blood flow and helps maintain a physiologic vaginal pH [36]. Over the longer term, lifestyle modifications avoiding smoking, limiting alcohol intake, and optimizing management of medical comorbidities can positively influence vaginal health in women with GSM, irrespective of breast cancer history or hormone receptor status [52].

*Hormonal treatments:* Women with moderate-to-severe GSM symptoms unrelieved by first-line nonhormonal measures may consider hormonal options to improve quality of life through a shared decision-making process, weighing the pros and cons, and involving their oncology team.

***Vaginal DHEA***: These inserts offer a potentially non-estrogenic therapeutic approach, but caution is advised by guidelines due to the metabolic conversion of DHEA to estrogen and testosterone, with slight elevation in serum testosterone levels but estrogen levels remaining in the postmenopausal range [4,47]. In women using AI therapy, although the aromatization from testosterone to estrogen is blocked, the long-term safety and survivorship data for oncologic outcomes is lacking. Thus, in clinical practice, oncologists and providers need to individualize this decision, balancing potential risks with quality-of-life considerations [30].

***Vaginal estrogen***: The use of LDVE in breast cancer survivors on AI therapy is nuanced. AIs suppress systemic estradiol to nearly undetectable levels due to inhibition of aromatization. However, unlike tamoxifen they do not block estrogen receptors, so even minimal elevation in systemic estradiol from LDVE is theoretically concerning. Across the few prospective and small randomized trials, low-dose local estrogen formulations such as the 10 µg estradiol vaginal tablet or 7.5 µg/day estradiol ring consistently improved vaginal dryness, dyspareunia, vaginal pH, and maturation index in breast cancer survivors using AIs, confirming their efficacy for GSM in this population [53]. Sensitive liquid chromatography mass spectrometry assays demonstrate that most women maintain serum estradiol concentrations within the postmenopausal range (<10–15 pg/mL), with transient or intermittent elevations reported in only a minority of participants [54,55]. In a recent meta-analysis of 118,659 breast cancer survivors, of which 6358 received LDVE and were followed up for durations ranging from 4 to 15 years, vaginal estrogen was not associated with a statistically significant increase in breast cancer recurrence overall (pooled RR 0.87; 95% CI 0.67–1.11) [44]. In the subgroup analysis of women concurrently using AIs, a non-significant higher recurrence risk was found with significant heterogeneity (RR 2.59, 95% CI 0.74–9.09; I^2^ = 95%), likely due to small number of studies, heterogeneity in AI exposure and residual confounding. There was no increase in breast cancer mortality (RR 0.60, 95% CI 0.18–1.95), and a lower all-cause mortality was seen in LDVE users (RR 0.80, 95% CI 0.75–0.86) [44]. The latter finding was attributed secondary to survivor selection benefit rather than a causal survival effect [44].

Given the absence of randomized or adequately powered observational studies directly assessing breast cancer recurrence or mortality risk in AI-treated women receiving local estrogen therapy, long-term oncologic safety remains uncertain [43,44,56]. Until definitive recurrence and survival data are available, the decision to use LDVE in women on AI needs to be an individualized, shared decision in coordination with the oncology specialist.

***Ospemifene***: Although preliminary data on effects of ospemifene for incidence of breast cancer or recurrence is favorable [49], ospemifene is not recommended for women with current or prior ER positive breast cancer because it has not been adequately studied in this population [4,28].

Outcome:

She proceeded with daily intravaginal DHEA 6.5 mg daily inserts, daily vaginal moisturizer use, silicone-based lubricants for intimacy, lidocaine 5% ointment for sensitive tissues over the introitus as needed, and pelvic floor relaxation exercises. A follow-up visit was scheduled in 3 months to assess the response to the suggested treatments, and for discussion of additional treatments, if needed.

**Case 2c:** GSM with a History of Triple (ER/PR/HER2)- Negative Breast Cancer.History of present illness
52-year-old postmenopausal, G3P2, femaleLMP at age 50Reports severe, progressive vaginal dryness and insertional dyspareuniaSymptoms have not improved with the use of vaginal lubricants, moisturizers and hypoallergenic approaches for vulvar careAttempts at penetrative sexual activity result in superficial introital tears and significant discomfortDescribes a complete loss of libido, which she attributes primarily to pain and fear of exacerbating her symptoms
Past history
Early-stage, right-sided triple-negative breast cancer (TNBC) five years ago, age 47○Underwent neoadjuvant chemotherapy○Followed by right breast lumpectomy and adjuvant radiation therapy○Genetic testing was negative for deleterious germline mutations○Has remained disease-free, with no evidence of recurrenceUp to date on breast cancer surveillance, colon, and cervical cancer screening
Medications & supplements
Atorvastatin 40 mg orally once dailyLansoprazole 30 mg orally once dailyIntranasal fluticasone 2 sprays each nostril once daily
Social history
Abstained from alcohol since her breast cancer diagnosisNonsmokerNo recreational drug use
Gynecologic examinationA mirrored examination was performed with patient participation. External genitaliaMarked labial thinning, loss of vulvar architecture, pallor of the vulvar vestibuleIntroitus appeared narrowed, with decreased elasticity and mild erythema at the posterior fourchetteVestibular tissue was pale, shiny, and atrophic, with scattered superficial fissures consistent with introital micro-tearsUrethral meatus was mildly erythematous, with a small caruncle notedSpeculum examinationPerformed using the smallest speculum and ample water-based lubricantFriable, smooth vaginal mucosa with loss of rugae and minimal secretionsCervix appeared flush with the vaginal apex, with no visible lesions or dischargeBimanual examination was limited due to discomfortHypertonic and tender pelvic floor muscles on palpationUnable to assess adnexa, uterus, or cervix

Management:

*Education and Counseling*: As discussed in cases 1 and 2a.

*Nonhormonal treatments*: Treatment with lubricants, moisturizers, topical lidocaine, hypoallergenic approach, pelvic floor relaxation exercises and dilator therapy (when appropriate), along with healthy lifestyle choices, remain the first-line treatments, as reviewed in cases 1–2b.

*Hormonal treatments*: In women with TNBC, the absence of estrogen receptor-mediated tumor biology allows greater flexibility in the management of GSM. Lack of adequate symptom response to nonhormonal options is a reasonable indication to consider either vaginal DHEA and low-dose vaginal estrogen treatment, with the choice guided by symptom severity, patient preference, and shared decision-making rather than a mandated therapeutic sequence.

***Vaginal estrogen***: Among hormonal options, local low-dose vaginal estrogen (LDVE) has the strongest evidence for efficacy and safety in TNBC. Multiple large observational studies, including the Danish nationwide cohort and population-based registries, have shown no significant increase in breast cancer recurrence or mortality among vaginal estrogen users [41,42,45]. Expert guidance notes that use of LDVE “may be less of a concern” in ER-negative survivors [42,45].

***Vaginal DHEA***: Survivorship data in women with TNBC for vaginal DHEA use are sparse, so guidelines advise case-by-case use, combined with oncology input.

***Ospemifene***: As noted in Case 2b, despite reassuring preliminary data of ospemifene on breast cancer incidence or recurrence, in the absence of long-term safety outcomes, ospemifene is not recommended for women with known or suspected breast cancer [4,48].

Outcome:

The patient was provided a prescription for Imvexxy 10 μg vaginal estradiol insert, to be used two times per week, in addition to hyaluronic acid-based vaginal moisturizer application three times per week, and advised to use silicone or water-based lubricant for sexual intimacy. A follow-up visit was arranged in 2–3 months for review of response to treatment. 

**Case 3:** GSM with a History of Urinary Urgency and Recurrent UTIs.History of present illness
77-year-old, postmenopausal, G4 P3 femaleLMP: At age 52, 25 years agoPresents for management of UTIs. She has experienced 4 UTIs in the past yearPrior urine cultures were positive for *E. coli*, and the most recent culture grew *E. coli* resistant to nitrofurantoin and trimethoprim-sulfamethoxazoleHistory of urinary incontinence, which has improved use of pelvic floor physical therapy, timed voiding strategies, and avoiding bladder irritantsHowever, she continues to experience urinary urgency and UTIsA recent cystoscopy showed no structural abnormalities
Past history
HypertensionHyperlipidemiaObesity, BMI 32Urinary incontinenceTubal Ligation, 36 years ago, age 41
Medications & supplements
Losartan 100 mg dailyRosuvastatin 10 mg daily
Social history
Alcohol: 3 drinks (5 oz wine glass per drink) per weekFormer tobacco use; quit 10 years agoNo recreational drug useMarried and in a supportive relationship with her husband of 50 years
Gynecologic examinationMirrored exam with patient participation performed. External genitaliaLabia appeared normal with some involution, folds preservedNo interlabial fissures or white lesionsPale vulvar vestibule with atrophic mucosaVaginal introital narrowing presentProminent urethral meatus with small urethral caruncle presentSpeculum examPale vaginal mucosa with loss of rugae with minimal secretionsBimanual examDeferred per patient request

Diagnosis:

Her clinical history coupled with the exam findings are suggestive of GSM, which can be a risk factor for recurrent UTIs in post-menopausal women.

Management:

*Education and Counseling:* For her recurrent UTIs (defined as ≥2 infections in 6 months or ≥3 in 12 months) [57], good perineal hygiene practices with avoidance of preservative- or paraben-containing products, vaginal spermicides, douching, or hyperosmolar lubricants or moisturizers were reviewed [12,58]. Importance of adequate hydration [57], healthy pelvic floor muscle function and treatment of urinary incontinence was also reviewed. She was reassured regarding the benign nature of the examination finding of urethral caruncle related to low estrogen, and conservative measures with sitz bath and vaginal estrogen were reviewed as treatment options [59].

*Hormonal treatments:* LDVE is an effective option to treat UTIs in postmenopausal women [60,61]. Estrogen deficiency during menopause can increase predisposition to recurrent UTIs by decreasing glycogen content in vaginal epithelial cells resulting in shifts in the normal vaginal microbiome. Estrogen deficiency can also result in thinning of the urethral mucosa and loss of compliance of the urethral seal [62]. LDVE can help restore the health of the urethral and vaginal tissues which can prevent the development of subsequent UTIs. In a retrospective analysis of 5638 of primarily postmenopausal patients, use of vaginal estrogen resulted in a 50% decrease in mean UTI incidence over 1 year of use [63]. These findings were confirmed in a meta-analysis of 324 patients from 5 RCTs comparing vaginal estrogen formulations to placebo [64]. Use of systemic estrogen therapy did not demonstrate similar benefit [64]. Several guidelines recommend the use LDVE for UTI prevention in women with GSM and recurrent UTIs [4,9]. Vaginal DHEA has been shown in one study to be effective for the prevention of UTIs, but the data is sparse for its use in UTI prevention [65].

*Nonhormonal treatments:*

***Methenamine***: Methenamine undergoes hydrolysis to formaldehyde in acidic urine (urinary pH < 6) and acts as a bacteriostatic agent at the urinary pH of ≤ 5.5. Adjunctive measures such as dietary acidification or vitamin C may be considered to acidify urine. Methenamine Hippurate 1 g twice daily has been demonstrated as a non-inferior strategy to antibiotic prophylaxis for recurrent UTIs in adult women from *E. coli*, enterococci and staphylococci, but not from proteus or pseudomonas because the alkaline urinary pH created by proteus and pseudomonas impairs the conversion of methenamine to formaldehyde. Methenamine thus offers a non-antibiotic alternative that avoids antimicrobial resistance. Methenamine is contraindicated in patients with renal or hepatic insufficiency and avoided in patients using sulfonamides due to formation of precipitates and crystals in combination with formaldehyde [66]. Longer term use of methenamine hippurate for recurrent UTIs requires shared decision-making as the safety data is limited to one year [60,67].

***Antimicrobial prophylaxis***: Antimicrobial prophylaxis may help reduce UTI risk compared to placebo [68]. The decision to use antimicrobial prophylaxis should be a shared decision that takes into account the benefits vs. adverse effects of the treatment with potential risk for antimicrobial resistance [60].

***Other products****:* Cranberry products and D-Mannose are commonly used in practice, but the evidence of their efficacy from clinical trials is mixed [60,67,69]. Recent meta-analyses of D-mannose have reported inconsistent and non-significant effects on recurrent UTI prevention, and substantial heterogeneity across trials (e.g., RR 0.44, 95% CI 0.18–1.11; I^2^ ~90%) [70]. Similarly, evidence on cranberry products shows modest reductions in UTI risk in some populations, but effect estimates vary widely due to differences in proanthocyanidin formulation, dosing, and study design, resulting in moderate heterogeneity in pooled analyses [71]. Despite these considerations, with the relative safety profile, addition of one or both of these agents to the treatment regimen is generally a reasonable approach. Use of energy-based treatments such as vaginal CO_2_ laser therapy or YAG for recurrent UTI symptoms is not recommended given the paucity of data for efficacy and safety [32,72].

Outcomes:

Estradiol 0.01% cream was initiated for management of GSM and prevention of recurrent UTIs in a dose of 1 g per application, applied with a gentle massage over the vulvar vestibule twice weekly. Concurrent use of nonhormonal strategies was emphasized and a follow-up visit was scheduled in 3 months.

## 4. Summary Points and Key Messages for Clinical Practice

Key Messages for Clinical Practice

1. GSM affects vulvovaginal, urinary, and sexual health and is highly prevalent yet underdiagnosed and undertreated. Clinicians need to proactively ask about GSM symptoms.
2. Clinicians should be able to counsel women on the selection of safe and effective vaginal lubricants and moisturizers, as well as adjunctive treatments, including pelvic floor physical therapy, in appropriate situations.
3. Clinicians should feel confident discussing and prescribing hormonal therapies for GSM incorporating patient preferences. Counseling should emphasize the distinction between systemic and local hormone therapies, underscoring the favorable safety profile and minimal systemic absorption of local treatments to help alleviate patient anxiety and improve adherence.
4. GSM can be managed effectively in breast cancer survivors, many of whom can use vaginal hormone therapies utilizing shared decision-making with their oncologist.
5. Vaginal estrogen therapies should be considered in women with GSM experiencing recurrent urinary tract infections or urinary urgency.

## 5. Conclusions

GSM is a common, progressive, and under-recognized condition, with significant physical, emotional and sexual health implications if left untreated. Stepwise therapy is recommended, starting with nonhormonal options such as high quality, hypoallergenic lubricants and vaginal moisturizers. Low-dose vaginal estrogen is the most effective and safe treatment for GSM. It is available in various formulations, and when used in appropriate doses, the systemic absorption is minimal. Vaginal DHEA and oral ospemifene are FDA-approved alternatives for the management of GSM. In breast cancer survivors, GSM treatments need to be individualized based on multiple factors, including estrogen receptor status and adjuvant therapy. Shared decision-making is recommended. Recurrent UTIs are common during the hypoestrogenic state of late menopause. Vaginal estrogen needs to be considered as an important part of the treatment for the prevention of recurrent UTIs in older women. GSM is a chronic and progressive condition, and ongoing treatment is required for maintaining symptom relief.

## Figures and Tables

**Table 1:** Characteristics, Composition and Clinical Considerations of Common Vaginal Lubricants and Moisturizers.

Product Type	Typical pH Range	Typical Osmolality (mOsm/kg)	Key Components	Mechanism/Key Effects	Advantages	Limitations/Cautions	Clinical Considerations/Ideal Use
	Lubricants						
Water-based lubricants (standard, hyperosmolar)	4.0–7.0	Often > 1000 (commonly 2000–6000)	Water, glycerin, glycerol, propylene glycol, parabens, preservatives	Provide short-term lubrication by coating mucosa and reducing friction	Widely available; inexpensive; condom-compatible	Hyperosmolarity can damage vaginal epithelium, increase BV and Candida risk; short duration	Choose glycerin-free, low-osmolality (<1200 mOsm/kg) for GSM; avoid in fertility or recurrent vaginitis
Water-based lubricants (iso-osmolar or nearly iso-osmolar)	4.0–5.5	~260–320 (physiologic)	Water, hydroxyethylcellulose, minimal glycerin, pH buffers	Mimic physiologic vaginal secretions; maintain mucosal integrity without osmotic stress	Gentle on tissues; minimal irritation; preserve lactobacilli; condom-safe	Slightly shorter duration than silicone-based; may dry faster	Ideal for GSM, sensitive mucosa, breast cancer survivors, fertility settings;
Silicone-based lubricants	6.0–7.0	200–500	Dimethicone, cyclomethicone, silicone polymers	Form long-lasting, non-absorptive hydrophobic film that reduces friction	Long-lasting; low osmolality; minimal irritation; condom-safe	Harder to wash off; may stain fabrics; not compatible with silicone sexual aids	Excellent for GSM; safe with latex condoms; ideal for mucosal sensitivity
Oil-based lubricants (natural or mineral oils, petroleum jelly)	5.0–8.0	200–1000	Natural oils, mineral oil, petrolatum	Provide occlusive barrier reducing water loss	Long-lasting; inexpensive	Degrade latex condoms; increase BV/Candida risk; limited safety data	Use only in non-condom users; avoid in infection-prone women or fertility attempts
Low-osmolality/‘fertility’ lubricants (e.g., Pre-Seed)	7.0–8.0	270–360	Hydroxyethylcellulose, glycerin-free polymers	Mimic cervical mucus and maintain sperm viability	Sperm-safe; iso-osmolar; minimal epithelial irritation	Shorter duration; higher cost	Preferred for conception and GSM with mucosal sensitivity
Glycerin-containing lubricants	4.0–7.0	>2000	Glycerin/glycerol, propylene glycol	Retain moisture via hygroscopic effect	Smooth texture; widely available	Promote BV/yeast; epithelial irritation; impair sperm motility	Avoid in GSM, fertility settings, or recurrent vaginitis
	**Moisturizers**						
Vaginal moisturizers (polycarbophil- or hyaluronic acid–based)	3.5–5.0	200–600	Water, polycarbophil, hyaluronic acid, lactic acid	Rehydrate and adhere to vaginal epithelium; lower pH to restore lactobacilli	Prolonged hydration for 2–3 days; safe for breast cancer survivors	Do not reduce friction during sex; require regular use	First-line nonhormonal therapy; apply every 1–3 days per severity
Hyaluronic acid–based moisturizers (subset)	3.8–4.5	200–500	Hyaluronic acid, buffering agents	Attract and retain water within mucosa; support epithelial repair	Improve elasticity, epithelial health, and comfort	Typically more expensive; need ongoing use	Useful for women avoiding hormones or seeking long-term hydration

**Table 2:** Low-Dose Vaginal Estrogen Therapy Options.

Name	Type	Daily Dose	How to Use	Serum Levels	Unique Considerations
Estradiol Vaginal Cream (0.01%) 0.1 mg/g vs. Premarin (Conjugated equine estrogen) vaginal cream 0.625 mg/g	Creams	0.5–1 g daily × 2 weeks, then 1–3 times/week	Apply with a massage over the vestibule/introitus and lower vagina, which can be done anytime of the day (preferred). Alternatively, apply intravaginally with applicator at night	Postmenopausal range with estradiol cream; varies by dose/assay Premarin levels not measurable	Allows targeted application to affected areas, with the option of gentle massage to enhance absorption while minimizing messiness. Manually measured (somewhat inexact) dosing
Estradiol Vaginal Tablet (10 μg)	Tablet	10 μg daily × 2 weeks, then twice weekly	Insert intravaginally with applicator	Typical serum ~4.6 pg/mL (range 3–11 pg/mL); ~1.14 mg systemic annually	Consistent dosing; widely used; among lowest systemic absorption
Estradiol Vaginal Insert (4 or 10 μg)	Softgel	Daily × 2 weeks, then twice weekly	Place intravaginally (finger or applicator)	No significant difference from placebo in systemic exposure	Premeasured dosing; convenient; lowest systemic absorption
Estradiol Vaginal Ring (7.5 μg/day release)	Ring	Releases 7.5 μg/day for 90 days	Insert in vagina; replace every 90 days	Typical serum ~8.0 pg/mL; ~2.74 mg systemic annually	Continuous dosing; convenient; stable serum levels; removable by patient

## Data Availability

No new data were created or analyzed in this study. Data sharing is not applicable to this article.

## Abbreviations

AIs, aromatase inhibitors; AUA, American Urologic Association; DHEA, dehydroepiandrosterone; ER, estrogen receptor; ET, estrogen therapy; FDA, US Food and Drug Administration; GSM, genitourinary syndrome of menopause; HER2, human epidermal growth factor receptor 2; ISSWSH, International Society for the Study of Women’s Sexual Health; LDVE, low-dose vaginal estrogen; LMP, last menstrual period; NAMS, North American Menopause Society (now The Menopause Society); PLISSIT, Permission, Limited Information, Specific Suggestion, and Intensive Therapy; PR, progesterone receptor; SERM, selective estrogen receptor modulator; TNBC, triple negative breast cancer; VTE, venous thromboembolism; Er: YAG, erbium-doped yttrium aluminum garnet; UTI, urinary tract infection.
